# Retracted: Effect of Stellate Ganglion Block Combined with Lidocaine at Different Concentrations for Preemptive Analgesia on Postoperative Pain Relief and Adverse Reactions of Patients Undergoing Laparoscopic Cholecystectomy

**DOI:** 10.1155/2022/9804601

**Published:** 2022-11-24

**Authors:** Computational and Mathematical Methods in Medicine

*Computational and Mathematical Methods in Medicine* has retracted the article titled “Effect of Stellate Ganglion Block Combined with Lidocaine at Different Concentrations for Preemptive Analgesia on Postoperative Pain Relief and Adverse Reactions of Patients Undergoing Laparoscopic Cholecystectomy” [1] due to concerns that the peer review process has been compromised.

Following an investigation conducted by the Hindawi Research Integrity team [2], significant concerns were identified with the peer reviewers assigned to this article; the investigation has concluded that the peer review process was compromised. We therefore can no longer trust the peer review process and the article is being retracted with the agreement of the Chief Editor.
